# 454-Pyrosequencing Analysis of Bacterial Communities from Autotrophic Nitrogen Removal Bioreactors Utilizing Universal Primers: Effect of Annealing Temperature

**DOI:** 10.1155/2015/892013

**Published:** 2015-09-03

**Authors:** Alejandro Gonzalez-Martinez, Alejandro Rodriguez-Sanchez, Belén Rodelas, Ben A. Abbas, Maria Victoria Martinez-Toledo, Mark C. M. van Loosdrecht, F. Osorio, Jesus Gonzalez-Lopez

**Affiliations:** ^1^Department of Civil Engineering, University of Granada, Campus de Fuentenueva, s/n, 18071 Granada, Spain; ^2^Institute of Water Research, University of Granada, C/Ramón y Cajal 4, 18071 Granada, Spain; ^3^Department of Biotechnology, Technical University of Delft, Julianalaan 67, 2628 BC Delft, Netherlands

## Abstract

Identification of anaerobic ammonium oxidizing (anammox) bacteria by molecular tools aimed at the evaluation of bacterial diversity in autotrophic nitrogen removal systems is limited by the difficulty to design universal primers for the *Bacteria* domain able to amplify the anammox 16S rRNA genes. A metagenomic analysis (pyrosequencing) of total bacterial diversity including anammox population in five autotrophic nitrogen removal technologies, two bench-scale models (MBR and Low Temperature CANON) and three full-scale bioreactors (anammox, CANON, and DEMON), was successfully carried out by optimization of primer selection and PCR conditions (annealing temperature). The universal primer 530F was identified as the best candidate for total bacteria and anammox bacteria diversity coverage. Salt-adjusted optimum annealing temperature of primer 530F was calculated (47°C) and hence a range of annealing temperatures of 44–49°C was tested. Pyrosequencing data showed that annealing temperature of 45°C yielded the best results in terms of species richness and diversity for all bioreactors analyzed.

## 1. Introduction

Anaerobic ammonium oxidizing (anammox) bacteria belong to the* Candidatus *Brocadiales order first described in 1999 [1]. Their ability to perform anaerobic ammonium oxidation has attracted the attention of many researchers due to the change it made for the understanding of the nitrogen cycle. They have been found as important bacteria for the ecology of nitrogen in oceanic environments [2–4] and have been proposed to play an important role in the nitrogen cycle at global scale [2]. Anammox bacteria have been identified in many natural and engineered environments such as marine sediments, agricultural soils, or wastewater treatment plants [5–7]. In addition, anammox bacteria are the basis for promising technologies aimed at removing nitrogen from wastewater. The autotrophic, anaerobic ammonium oxidation has been utilized for the set-up of several technologies for nitrogen removal, such as partial nitritation/anammox, DEMON, OLAND, or CANON [8]. Autotrophic nitrogen removal technologies account for several advantages over traditional total nitrification-denitrification processes, such as the lesser bioreactor volume required, lower biomass production, or saving costs in aeration and carbon source requirements [9–11]. Thus, anammox bacteria have become of relevance in both natural and engineered systems, and more research about the ecology of the ecosystems where they develop is expected in the following years.

PCR-based molecular biology techniques such as qPCR quantification and high-throughput pyrosequencing are powerful tools for the estimation of the abundance and diversity of microorganisms in natural and engineered ecosystems [12, 13]. Most frequently, anammox-specific primers have been applied for PCR-based evaluation of the occurrence and diversity of these organisms [12, 14–18]. The reason under this common practice is that universal primers targeting the whole* Bacteria* domain tend to amplify poorly the 16S rRNA gene of anammox due to their not very high identity (<87.1%) [19, 20]. In this sense, when universal primers are used PCR amplification of some anammox phylotypes might be missing, with the consequent loss of fundamental information on the microbial diversity of the system. Nevertheless, the use of anammox-specific primers is not sufficient for a complete understanding of microbial ecosystems, given that other bacteria are not taken into account.

In this research, several universal primers and annealing temperature conditions for PCR amplification were tested in order to achieve the best combination possible for its use on the metagenomic analysis of ecosystems, such as autotrophic nitrogen removal bioreactors. After an* in silico* testing, a universal primer was selected for the best coverage of 16S rRNA genes of the domain* Bacteria *including anammox (*Planctomycetes*). Using this primer, bacterial diversity in samples from five different lab-scale and full-scale autotrophic nitrogen removal bioreactors (see Table S1 in Supplementary Materials available online at http://dx.doi.org/10.1155/2015/892013) were analyzed by high-throughput pyrosequencing, using six different annealing temperatures. Based on the results obtained, a robust method for the evaluation of the bacterial diversity in ecosystems where anammox bacteria are of importance is proposed.

## 2. Materials and Methods

### 2.1. Bioreactors

Five different autotrophic nitrogen removal bioreactors which represent the main existing anammox technologies were sampled in the analysis (Table S1). Two of them were bench-scale models and the other three were full-scale plant bioreactors. The bench-scale models analyzed in the study were named Lab MBR and Low Temperature CANON. Lab MBR is a membrane bioreactor (MBR) anammox system and Low Temperature CANON is a CANON system operated at 15°C. Both bioreactors were built in Netherlands in 2010 and 2009, respectively, and were fed synthetic wastewater.

The full-scale bioreactors sampled in the study were located at three cities in Netherlands, Apeldoorn, Olburgen, and Rotterdam, and were named A, N, and R, respectively. Bioreactor A is a DEMON system built in 2010 which treats reject water from anaerobic digester. Bioreactor N is a CANON process treating sewage from a potato processing factory built in 2009. Bioreactor R is the anammox reactor in a two-step anammox plant constructed in 2002 and treats reject water from anaerobic digester.

### 2.2. Sampling

Five samples (200 mL) were taken from five evenly distributed points within each bioreactor volume. The procedures for sampling and pretreatment for DNA extraction followed those described by Ni et al. [16]. Biomass was separated from the collected wastewater by centrifugation at 3500 rpm for 10 minutes at room temperature in a Kokusan H-103N series apparatus (Kokusan Enshinki Co., Ltd., Tokyo, Japan). Pelleted biomass was stored at −20°C before DNA extraction.

### 2.3. *In Silico *Primer Evaluation

The* primer pair* 530F (5′-GTGCCAGCMGCNGCGG)-1100R (5′-GGGTTNCGNTCGTTG), described by Dowd et al. [21], was selected after an* in silico *analysis of several universal primers targeting the 16S rRNA gene and commonly used in earlier literature. The primers were tested by correlating the accession numbers of the matching sequences, using the Probe Match function of the Ribosomal Data Project (RDP) (http://rdp.cme.msu.edu/probematch/search.jsp) (see Supplementary Material). Moreover, to double check the performance of the selected primer set over the* Planctomycetes* phylum, an extra* in silico* analysis study using the SiLVA Test Probe tool was done (http://www.arb-silva.de/search/testprobe).

### 2.4. *In Silico *Calculation of Optimum Annealing Temperature

Calculation of optimum annealing temperature for primer 530F was done following the expression for the optimum salt-adjusted annealing temperature of a primer developed by Rychlik et al. [22–25]. For calculation purposes, combined concentration of Na^+^ and K^+^ was taken as 50 mM.

### 2.5. DNA Extraction, PCR Amplification, and Pyrosequencing

300 mg of pelleted biomass for each centrifuged sample was used for DNA extraction using the Fast DNA SPIN Kit for Soil and the Fast-Prep24 apparatus (MP Biomedicals, Solon, OH, USA) following the instructions given by the manufacturer. DNA extracts from samples collected in the same bioreactor were merged into a pool. The DNA pool of each bioreactor was divided into 6 subsamples with equal volume for further pyrosequencing analysis.

The primer pair 530F-1100R [21] was used to amplify 500 bp of the 16S rRNA gene of* Bacteria*, encompassing the V4-V5-V6 hypervariable regions. Research and Testing Laboratory (Lubbock, Texas, USA) proceeded with pyrosequencing following the procedure described by Dowd et al., 2008 [21], using the Roche 454 GS-FLX+ apparatus. Amplification of the six subsamples within the same bioreactor DNA pool was assayed under the same PCR conditions but at different annealing temperatures (44 to 49°C), yielding a total of 30 different pyrosequencing datasets in the five different technologies. In this sense the PCR conditions for pyrosequencing were the following: preheating step at 94°C for 3 minutes; 32 cycles at 94°C for 30 seconds, 44–49°C for 40 seconds, and 72°C for 1 minute; elongation at 72°C for 5 minutes.

### 2.6. Pyrosequencing Postrun Analysis

Elimination of poor-quality end reads from pyrosequencing raw data was done by quality trimming based on quality scores. USEARCH [26] was then used to generate seed sequences to which quality trimmed reads were clustered within a 4% divergence threshold. This procedure eliminates sequences that fail to encounter similar enough reads. Chimera detection was developed using the* de novo* method implemented in UCHIIME [27] over clustered quality trimmed sequences collected during the previous step. Denoising was then conducted for elimination of bad sequences and correction of base pair errors. Following denoising, a quality control screening was conducted. Quality criteria taken were the following: (1) failed sequence reads, (2) sequences with low quality tags, and (3) sequences that are shorter than half the expected amplicon length or 250 bp, whichever the shortest. Sequences that could not meet the defined quality criteria were eliminated. Reads that passed the quality screening control were then clustered for phylogenetic identification into 0% divergence using USEARCH [26]. The Kraken BLAST software (http://ccb.jhu.edu/software/kraken/) [28] was utilized to provide a seed sequence for each phylogenetic identification cluster from a high-quality database derived from the NCBI GenBank database. Based on BLASTN+ identity, sequences were affiliated to distinct taxonomic levels as follows: (1) at OTU level if divergence was less than 3%, (2) at genus level if divergence was in the range 3–5%, (3) at family level if divergence was 5–10%, (4) at order level if divergence was 10–15%, (5) at class level if divergence was 15–20%, and (6) at phylum level if divergence was 20–23%. Sequences that did not encounter queried sequences with less than 23% divergence were discarded.

### 2.7. Rarefaction Curves

Rarefaction curves for each sample were calculated using the aRarefactWin software developed by S. Holland (University of Georgia, Athens; http://strata.uga.edu/software/). For the purpose of microbial community analysis, rarefaction curves of samples belonging to the same bioreactor were interpolated to the lowest reads count among all of them and extrapolated to the highest reads count among all of them. Extrapolation of rarefaction curves was done following the mathematical model described in Colwell et al. [29].

### 2.8. Heat Maps

For each bioreactor, a heat map was generated defining the differences of community structure on the basis of the annealing temperatures selected for PCR. The heat maps were based on the relative abundance of OTUs > 1% relative abundance.

### 2.9. Cluster Analysis (CA)

Three different types of cluster analysis (CA) were performed over the samples: “analysis A” including all OTUs and using a non-phylogeny-dependent method; “analysis B” including only OTUs with >1% relative abundance and using a non-phylogeny-dependent method; and “analysis C” including only OTUs with >1% relative abundance and using a phylogeny-dependent method. Non-phylogeny-dependent A and B analysis were based on Bray-Curtis dissimilarity and carried out with the Vegan package v.2.0 implemented on the statistical software R-Project v.2.15.1 [30]. The software Fast UniFrac [31] was utilized for the phylogeny-dependent C analysis, following instructions given by the developers in the software tutorial (http://unifrac.colorado.edu/). For this purpose, reference trees were constructed for each bioreactor, using the MEGA 6.0 software [32]. Relative abundances of each of the OTUs were used as weight for the analysis.

### 2.10. Principal Coordinates Analysis (PCoA)

Principal coordinates analysis (PCoA) of samples coming from the same bioreactor was done based on a phylogeny-dependent method. OTUs with relative abundance >1% were selected for the study. For each bioreactor, a reference tree with selected OTUs was constructed using MEGA 6.0 software. Utilizing reference trees, phylogeny-based PCoA was conducted using Fast UniFrac software [31], following the instructions given in the software tutorial (http://unifrac.colorado.edu/).

### 2.11. Hill Diversity Indices

Hill diversity indices of order 1 (Shannon index) and order 2 (Simpson index) were calculated for each sample using the Vegan package v.2.0 implemented on the statistical software R-Project v.2.15.1 [30].

## 3. Results and Discussion

### 3.1. *In Silico* Primer Evaluation

The design of a primer that covers all species within the domain* Bacteria* is impossible [33]. Therefore, when a primer is chosen for a bacterial community analysis, one has to accept that no total coverage could be found. For this reason, potential coverage of a primer is of primary importance in order to decide the best option for the analysis of microbial ecology of a natural ecosystem. Results of the* in silico* search for coverage of bacterial species of nine widely used universal primers within the RDP database are displayed in Table 1. Only results for the phylum* Planctomycetes* and the complete* Bacteria* domain are shown. Results for all other phyla can be seen in Table S2. The results of the* in silico* analysis showed that primer 530F offered the best coverage of species for both the* Bacteria* domain and the phylum* Planctomycetes*. Primer 530F was able to discern 76.01% of the total bacterial species known to date. The closest follower was primer 519R, with 8% less total coverage. With regard to* Planctomycetes*, primer 530F covered 75.58% of species belonging to this group, while the second best (910R) covered 53.70%.

Coverage of all phylotypes of anammox bacteria has been reported as very difficult if a universal primer is utilized [19, 20].* In silico *analysis of the coverage of all* Candidatus *Brocadiales microorganisms by primer 530F within the SiLVA database is summarized in Table S3. We found that primer 530F targeted the 16S rRNA genes of all the anammox* Candidatus* species known to date yielding a minimum of 90% perfect matches to sequences filed in the database. Thus, it can be stated that primer 530F is the best option for the metagenomic analysis of autotrophic nitrogen removal bioreactors.

For the purposes of pyrosequencing, reverse primer 1100R was taken, following the literature that has utilized primer 530F for pyrosequencing analysis [21].

### 3.2. *In Silico *Optimum Annealing Temperature Calculation

Following the expression given by Rychlik et al. [22] for the salt-adjusted optimum melting temperature of primer 530F with combined N^+^ and K^+^ concentration of 50 mM, as utilized by other authors [23–25], optimum melting temperature for primer 530F was 47°C. For the testing of different annealing temperatures an interval around optimum annealing temperature calculated was covered, ranging from 44°C to 49°C.

### 3.3. Rarefaction Curves

For each bioreactor, original rarefaction curves, interpolated ones to lowest number of reads within samples, and extrapolated ones to the highest number of reads within samples were generated and are shown in Figure S1. It can be seen that annealing temperatures of 44°C offer lower species richness than all the other annealing temperatures tested.

### 3.4. Diversity and Relative Abundance of Bacterial Species

**Figure 1:**
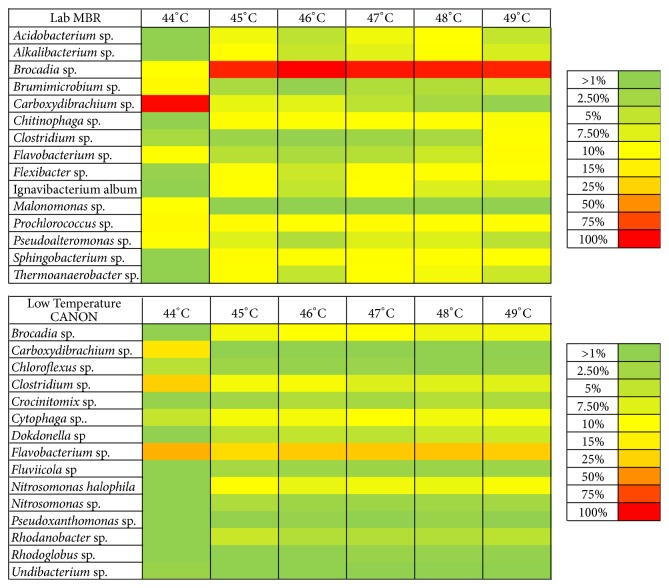
Heat maps of OTUs > 1% relative abundance in lab-scale bioreactors LAB MBR and Low Temperature CANON.

**Figure 2:**
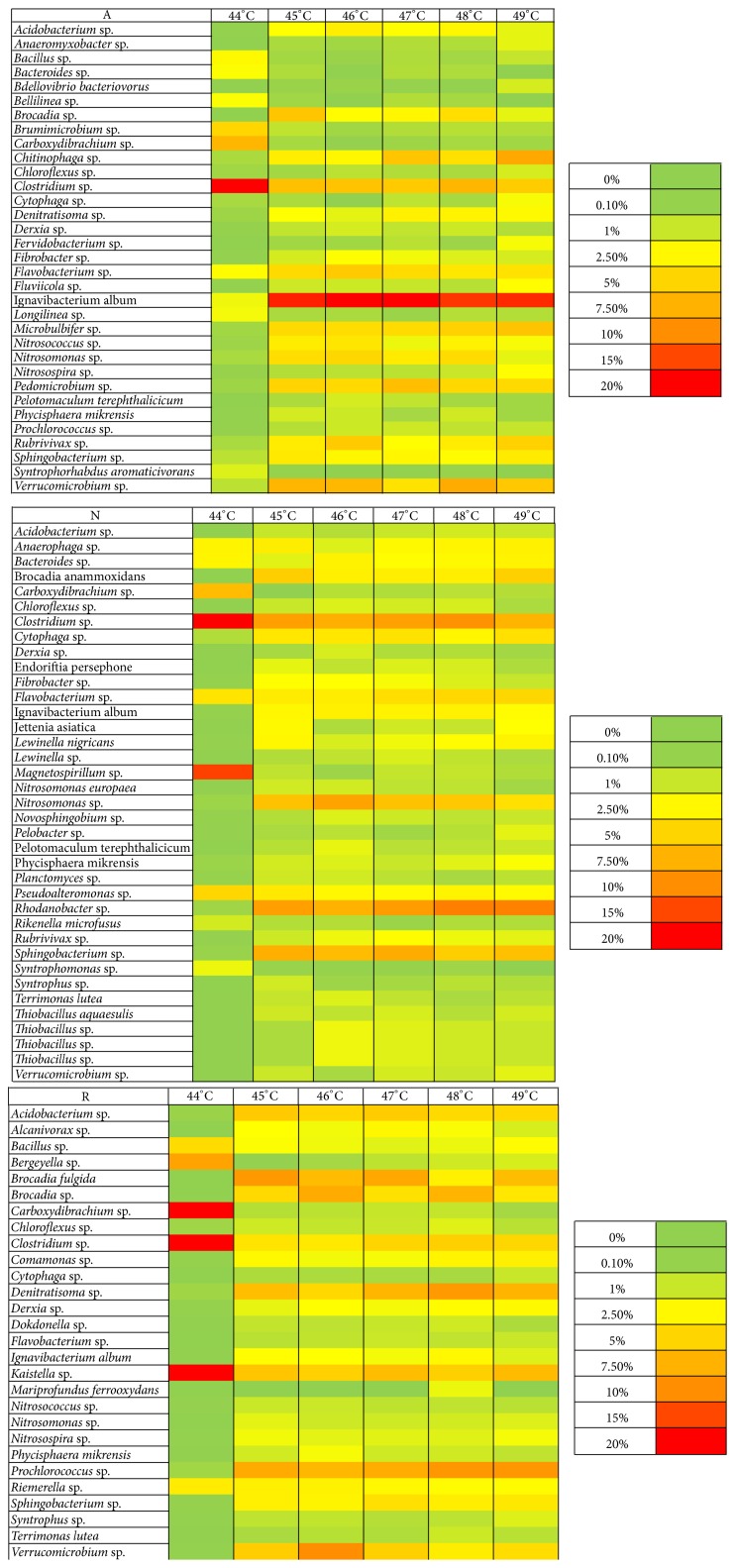
Heat maps of OTUs > 1% relative abundance in full scale bioreactors Appeldorn (A), Olburgen (N), and Rotterdam (R).

Heat maps were generated for each bioreactor taking into account only the OTUs with >0.1% relative abundance as shown in Figures 1 and 2. Annealing temperature of 44°C showed a pattern that differed from those generated at all other temperatures in all bioreactors. Interestingly, at 44°C bacteria belonging to the* Candidatus *Brocadiales order (*Candidatus *Brocadia anammoxidans,* Brocadia fulgida,* and* Brocadia* sp.) showed a much lower relative abundance than that at all the other annealing temperatures tested for every bioreactor. Particularly, for the Lab MBR bioreactor, which stands as a highly enriched (>90%) [34] culture of Brocadiales, the relative abundance found was 1.27% when 44°C was the annealing temperature, while at the other temperatures assayed it fell in the 75–86% range.

Amplification of partial 16S rRNA genes of bacteria other than anammox was also influenced by the annealing temperature selected. In the case of the Lab MBR, the relative abundance of copies of* Carboxydibrachium *sp.was of 84.5% at 44°C, much higher compared to the other annealing temperatures tested. The inability to capture any* Candidatus *Brocadiales phylotypes in all bioreactors, with one of those being a high enrichment of these bacteria, when annealing was performed at 44°C is related to a consistent error occurring during the PCR procedure of primer 530F. In this sense, it is possible that annealing at 44°C hinds the relative abundance of* Candidatus *Brocadiales bacteria under other bacterial species such as* Carboxydibrachium *sp. in the different pyrosequencing samples from the five bioreactors analyzed.

The results described here demonstrated that an annealing temperature of 44°C was inappropriate to analyze the bacterial community structure of the bioreactors sampled in the study, due to a severe underestimation of the relative abundance of Brocadiales.

Results of bacterial diversity obtained for bioreactors Lab MBR and Low Temperature CANON were further analyzed to check the performance of primer 530F for the evaluation of anammox diversity. Diversity of anammox bacteria in bioreactors Lab MBR and Low Temperature CANON was studied before by other authors utilizing different molecular biology techniques, such as FISH or qPCR [35, 36]. In these studies, it was concluded that Candidatus* Brocadia fulgida* was the only anammox bacteria in both Lab MBR and Low Temperature CANON bioreactors. In the present study, Candidatus* Brocadia *sp. was the only anammox identified in Lab MBR bioreactor, while in the Low Temperature CANON bioreactor, pyrosequencing with primer 530F retrieved partial 16S rRNA genes affiliated to four different anammox phylotypes:* Candidatus *Brocadia anammoxidans,* Candidatus Brocadia fulgida*,* Candidatus Brocadia* sp., and* Candidatus *Jettenia asiatica, all of them represented in low relative abundance with the exception of* Candidatus Brocadia* sp. Sequences for the OTUs identified as* Candidatus Brocadia* sp. in both Lab MBR and Low Temperature CANON bioreactors (305 and 289 nucleotides, resp.) shared 99% similarity in 100% query cover with four* Candidatus Brocadia fulgida* sequences found in the BLASTN database (JQ864319.1, JQ864321.1, JQ864322.1, Zheng & Zhang, unpublished, and JX243455.1 [36]). Thus, it can be said that universal primer 530F can express in a consistent way the diversity of anammox bacteria found in autotrophic nitrogen removal bioreactors.

Failure to find* Candidatus *Brocadiales sequences at annealing temperature of 44°C can be related to a bias of PCR procedure. The relative abundance of* Brocadia *sp. and* Carboxydibrachium *sp. along with their ratio for the bioreactor Lab MBR can be seen in Table 2. The* Carboxydibrachium *sp./*Brocadia *sp. ratio abruptly increases when dropping from 45°C to 44°C, while it stabilizes at temperatures of 45°C and higher. In accordance with our results, some studies have found that a small percentage of OTUs in chicken caecal samples suffered from distortions in relative abundance as their bacterial community structure was studied through RT-PCR at different annealing temperatures, even though the entire microbial community structures were not subjected to major changes [37]. Also, changes in amplification of* Vibrio vulnificus* by RT-PCR at different annealing temperatures have been found [38]. Mismatches of primers with the targeted region of the DNA templates are thought to be the cause of differences in the estimation of the relative abundance of bacterial species in environmental samples. A higher number of mismatches in the targeted region lead to a higher bias of the relative abundance of certain OTUs. Among other factors, the annealing temperature is thought to cause mismatch of primers during PCR processes [33]. In this way, low temperatures with respect to optimum have been related to the proliferation of amplicons with mismatches [39, 40]. On the other hand, it has been shown that high annealing temperatures with respect to optimum tend to increase the ratio of amplification of one-mismatch to no-mismatch sequences [41, 42]. Regardless of the diversity of opinions, knowledge of the factors that intervene in the differences of relative abundance of OTUs at different annealing temperatures is still incomplete; hence, primer mismatches may not be the only cause driving these changes [37].

### 3.5. CA and PCoA

CA a) and b) are shown in Figure S2. When the similarity of all OTUs identified was compared, there were no relevant differences between results generated by PCR at the different annealing temperatures assayed (CA a)). However, CA b), which analyzes only OTUs with >1% relative abundance, clearly shows significant differences between the results generated at 44°C compared to the rest of annealing temperatures tested. This is in accordance with the heat maps generated for the bioreactors sampled (Figure 1). This implies that Bray-Curtis dissimilarities between annealing temperatures are less pronounced when samples are studied to their full sampling depths.

Phylogeny-dependent CA c) for each bioreactor is shown in Figure S3. Similarity between samples varies depending on the bioreactor analyzed. Samples from lab-scale bioreactors showed a higher phylogeny-based similarity for all the annealing temperatures tested. No significant differences at 80% similarity were found in bioreactor Low Temperature CANON, and three clusters were formed at 90% similarity. For bioreactor Lab MBR there were no significant differences at 70% similarity, but three clusters were generated at 80% and 90% similarity. Full-scale bioreactors showed lower phylogeny-based similarity, with significant differences at 70% for bioreactors N and R and sequences grouped in five or six clusters at 90% similarity in all cases. This leads to the assumption that differences in bacterial communities obtained at different annealing temperatures are more pronounced in more complex ecosystems.

Phylogeny-dependent PCoA 2D views are offered in Figure S4. PCoA was done to double check the results obtained by CA of samples. Phylogeny-dependent PCoA showed that 44°C tended to discriminate from the other annealing temperatures in terms of microbial community structure explanation. On the other hand, samples annealed at 45°C and 48°C seemed to be related in terms of microbial community structure. As also proven by cluster analysis, the differences between various annealing temperatures were found more pronounced as the complexity of the bacterial communities increased.

Some authors have reported that differences in annealing temperature do not significantly affect the microbial community structure of chicken caecal samples [37]. In our case, differences in microbial community structure at both full sampling depth and at >1% OTUs were confirmed.

### 3.6. Species Richness and Hill Diversity Indices

Values for individuals computed and original, interpolated and extrapolated species richness are summarized in Table 3. Rating for performance of the different annealing temperatures based on species richness is shown in Table 4. Differences in species richness are accounted at the original sequencing depth, interpolated to the lowest number of reads per bioreactor, and extrapolated to the highest number of reads per bioreactor. Even though species richness comparison cannot be performed at different sequencing depths, methods for interpolation and extrapolation of rarefaction curves can precisely estimate species richness diversity of samples with different number of reads, making comparison between them possible [29].

Differences in species richness and number of reads obtained at each annealing temperature can be appreciated. As a main trend, 44°C is the temperature that offered the highest number of reads, followed by 45°C and 46°C. It has been suggested that annealing temperatures lower than the optimum increase the number of PCR products generated [43]. The lowest number of reads was obtained at 49°C. 48°C was the temperature generating a higher interpolated species richness, while at 44°C a poorer performance was observed, and 44°C was also the worst among all annealing temperatures regarding original species richness. Extrapolated species richness of samples was higher at 45°C, followed by 48°C.

In conclusion, 45°C is the annealing temperature showing the best performance regarding the expression of species richness of bacterial communities in the bioreactors studied, while annealing at 49°C offers a worse performance taking into account the low number of reads consistently obtained.

Hill diversity indices of first order (Shannon index) and second order (Simpson index) have been defended as the most robust method for comparison of bacterial assemblages from natural ecosystems [31]. Hill diversities of first order and second order for each sample are shown in Table 5. Ratings for the comparison of each annealing temperature based on the indices can be seen in Table 6. Both indices showed the same quality pattern, with 45°C being the highest value followed closely by 49°C and 48°C. Once again, 45°C was the best annealing temperature for capturing diversity of bacterial communities inside the bioreactors analyzed.

No significant differences in species richness and evenness of chicken caecal samples studied through RT-PCR have been reported [37]. Nevertheless, other authors have shown that differences in annealing temperature lead to different bonding of primer and targeted region of genetic templates, having an impact over ecological parameters of environmental samples. This is caused by enhanced amplification of certain strains, which deviates relative abundance of these species, or by nonspecific bonding, which increases the microbial diversity recorded on the samples [39, 44]. In our case, small differences in annealing temperature changed species richness, effective number of counts, and Hill diversities within the samples. Samples processed at 44°C accounted for the highest number of reads but surprisingly also contained the lowest species richness and diversity. This shows that annealing temperatures of 44°C reduced the estimation of the diversity of the system. Reduced diversity of samples analyzed at 44°C may rest on the fact that the primer is subjected to much more stringent bonding than that at the other annealing temperatures, therefore generating many sequences of a low number of species. All other annealing temperatures can capture the diversity of the ecosystem studied in a consistent fashion. 46°C offers a lower diversity quality values in comparison with the others.

In terms of species richness and diversity, 44°C offers poor results compared to the others, but it can be said that annealing temperatures ranging from 45 to 49°C offer good results. Nevertheless, 45°C stands as the optimal annealing temperature of all those tested due to superior species richness and diversity indices values. Interestingly, it yields better results in species richness and diversity than the* in silico* calculated salt-adjusted optimum for the primer. As suggested by Hecker and Roux, 1996 [43], annealing temperatures above and down the optimum annealing temperature theoretically calculated for a given primer increase number of reads obtained and specificity of PCR products. In this case, annealing temperature at 45°C gives better results, in terms of species richness and diversity, than the optimum salt-adjusted annealing temperature for the primer utilized.

## 4. Conclusions

The superior coverage of primer 530F with respect to other popular universal primers was indicated through* in silico* testing. The ability of primer 530F to target the majority of known anammox phylotypes was also demonstrated by* in silico *testing. Therefore, primer 530F stands as the best universal primer available for the metagenomic analysis of microbial communities where anammox bacteria are expected to develop important ecological functions.

The 30 pyrosequencing analysis showed that the annealing temperature produces a severe effect over the microbial community structure discerned through pyrosequencing. Strong bias in the identification of anammox species in particular at annealing temperature of 44°C was observed in all the pyrosequencing samples for each of the five autotrophic nitrogen removal technologies. The six different annealing temperatures analyzed in the study showed different microbial community structure compositions as proved by phylogeny-based and non-phylogeny-based CA and PCoA. Differences among annealing temperatures could also be observed with respect to the number of individuals sequenced and species richness with 45°C being the best in these terms. In this sense, annealing temperatures of 45°C demonstrate good coverage of total bacteria and anammox species, high number of reads, the highest species richness, and the highest diversity indices values. Therefore, results show that autotrophic nitrogen removal bioreactor bacterial community analyses including anammox bacteria can be done using the universal primer 530F with annealing temperature of 45°C. Under these conditions, this procedure is a good approach for the bacterial diversity study in autotrophic nitrogen removal technologies using pyrosequencing methods.

## Supplementary Material

Table S1 shows main operational parameters of the five bioreactors analyzed in this study.
Table S2 offers the results of the in silico primer testing using RDP database for all bacterial phyla.
Table S3 shows the in silico calculated coverage of anammox bacteria species by primer 530F using the RDP database.
Figure S1 shows the original, interpolated and extrapolated rarefaction curves of the different samples at different annealing temperatures for all bioreactors sampled in the study.
Figure S2 provides the Bray-Curtis based cluster analysis of samples at different annealing temperatures for all bioreactors sampled: a) accounting for all OTUs, and b) of >1% OTUs.
Figure S3 shows the phylogeny-dependent cluster analysis of samples at different annealing temperatures for all bioreactors sampled.
Figure 4 shows the phylogeny-based principal coordinates analysis of all annealing temperatures tested for each bioreactor sampled.

## Figures and Tables

**Table 1:** *In silico* evaluation of primer coverage of species in the *Bacteria* domain and *Planctomycetes* specific.

Phylum	27F	519R	530F	787R	910R	1064R	1392R	1492R

*Planctomycetes *	17.82%	23.89%	75.58%	6.17%	53.70%	39.88%	33.25%	6.59%

Total	**11.58%**	**68.47%**	**76.01%**	**59.45%**	**56.53%**	**54.52%**	**22.08%**	**4.08%**

**Table 2:** Relative abundances of *Brocadia *sp., *Carboxydibrachium *sp. and the ratio of relative abundances *Carboxydibrachium *sp. to *Brocadia *sp. for the Lab MBR reactor.

Lab MBR						
	44°C	45°C	46°C	47°C	48°C	49°C
Species						
*Brocadia* sp. (%)	1.04	75.15	86.76	79.51	77.68	75.77
*Carboxydibrachium *sp. (%)	84.50	0.81	0.77	0.44	0.26	0.13
*C.* sp./*B.* sp.	81.34	0.01	0.01	0.01	0.00	0.00

**Table 3:** Values for individuals computed, original species richness *S*(est), interpolated species richness *S*(int), and extrapolated species richness *S*(ext).

	Temperature	Individuals (computed)	*S*(est)	*S*(int)	*S*(ext)
Lab MBR (T2)	44°C	11646	42	34.8	42.99
	45°C	16895	148	85.6	148.4
	46°C	17336	117	67	117
	47°C	3162	78	76.8	85.67
	48°C	5883	100	79.2	108.9
	49°C	3024	105	105	117.3

Low Temperature CANON (TC)	44°C	11438	61	30.1	38.65
	45°C	14362	179	100.3	179
	46°C	9545	155	94.4	163.1
	47°C	4145	119	99.5	131.6
	48°C	5068	133	99.3	147.9
	49°C	2394	87	87	95.53

Apeldoorn DEMON (A)	44°C	9226	78	29.3	78.76
	45°C	9924	161.7	45.1	161.7
	46°C	654	73	55.6	79.11
	47°C	2192	141	66.5	156.9
	48°C	8206	258	68	265.2
	49°C	321	60	59.9	68.55

Olburgen CANON (N)	44°C	17193	87	41.9	87
	45°C	2893	198	134.2	218.3
	46°C	947	141	140.9	160.7
	47°C	3180	205	135.7	221.9
	48°C	2462	197	139.2	217.1
	49°C	1905	174	133.8	192.6

Rotterdam 2-stage anammox (R)	44°C	12977	81	43.2	81
	45°C	5183	211	147.3	228
	46°C	3879	188	143.5	205.2
	47°C	4120	198	151.6	212.3
	48°C	1679	143	143	159.6
	49°C	1791	145	142.3	159.7

**Table 4:** Ratings for the comparison of species richness for samples from different bioreactors at each annealing temperature. Higher mean ratio is related to higher performance of the annealing temperature.

Temperature	Mean ratio individuals	Mean ratio *S*(int)	Mean ratio *S*(ext)
44°C	89.36%	32.89%	31.00%
45°C	72.36%	88.05%	91.86%
46°C	41.79%	86.87%	72.43%
47°C	24.22%	93.29%	76.70%
48°C	37.09%	93.51%	84.76%
49°C	12.49%	92.73%	63.01%

**Table 5:** Values for the Shannon index and the Simpson index of all samples analyzed in the study.

	Temperature	Shannon index	Simpson index
Lab MBR (T2)	44°C	1.559179	0.4866806
	45°C	1.657458	0.5265754
	46°C	0.9898754	0.2964455
	47°C	1.398619	0.4398892
	48°C	1.536843	0.480206
	49°C	1.665004	0.4945622

Low Temperature CANON (TC)	44°C	2.02004	0.7847361
	45°C	3.018484	0.9061173
	46°C	2.85764	0.8814217
	47°C	2.86951	0.8790168
	48°C	2.860865	0.8741682
	49°C	2.871034	0.8860556

Apeldoorn DEMON (A)	44°C	2.297351	0.6694541
	45°C	3.561685	0.9399574
	46°C	3.286833	0.928345
	47°C	3.51128	0.9295173
	48°C	3.651016	0.944623
	49°C	3.366082	0.9407517

Olburgen CANON (N)	44°C	2.959167	0.8714149
	45°C	3.979032	0.9632393
	46°C	4.039	0.9667856
	47°C	4.021377	0.9636953
	48°C	4.015465	0.9615441
	49°C	3.965338	0.9629424

Rotterdam 2-stage anammox (R)	44°C	2.497644	0.8499261
	45°C	3.910746	0.9644197
	46°C	3.87392	0.9619344
	47°C	3.903737	0.9636846
	48°C	3.865236	0.9630658
	49°C	3.902146	0.9643079

**Table 6:** Rating for the comparison of species diversity for samples from different bioreactors at each annealing temperature. Higher mean ratio is related to higher performance of the annealing temperature.

Temperature	Mean ratio Shannon index	Mean ratio Simpson index
44°C	72.12%	85.63%
45°C	99.12%	99.83%
46°C	88.64%	90.32%
47°C	94.92%	95.71%
48°C	97.07%	97.40%
49°C	97.14%	98.24%
