# Supplementary material for: 454-Pyrosequencing Analysis of Bacterial Communities from Autotrophic Nitrogen Removal Bioreactors Utilizing Universal Primers: Effect of Annealing Temperature
Source: Biomed Res Int. 2015 Sep 3;2015:892013. doi: 10.1155/2015/892013 (PMC4573432; doi:10.1155/2015/892013)
Supplement: Supplementary file 1 — Table S1 shows main operational parameters of the five bioreactors analyzed in this study. Table S2 offers the results of the in silico primer testing using RDP database for all bacterial phyla. Table S3 shows the in silico calculated coverage of anammox bacteria species by primer 530F using the RDP database. Figure S1 shows the original, interpolated and extrapolated rarefaction curves of the different samples at different annealing temperatures for all bioreactors sampled in the study. Figure S2 provides the Bray-Curtis based cluster analysis of samples at different annealing temperatures for all bioreactors sampled: a) accounting for all OTUs, and b) of >1% OTUs. Figure S3 shows the phylogeny-dependent cluster analysis of samples at different annealing temperatures for all bioreactors sampled. Figure 4 shows the phylogeny-based principal coordinates analysis of all annealing temperatures tested for each bioreactor sampled. [file 892013.f1.pdf]

Phylum	27F	519R	530F	787R	910R	1064R	1392R	1492R
Proteobacteria	14.12%	73.40%	75.38%	67.26%	57.44%	49.16%	28.95%	5.74%
Firmicutes	11.71%	79.74%	82.96%	74.08%	69.07%	68.65%	18.63%	3.06%
Actinobacteria	5.43%	62.01%	88.32%	61.73%	72.60%	77.09%	15.40%	3.88%
Bacteroidetes	12.53%	68.38%	69.78%	60.81%	47.97%	46.38%	20.29%	2.80%
unclassified_Bacteria	14.25%	32.01%	48.71%	23.48%	26.31%	29.02%	18.11%	2.97%
Acidobacteria	12.75%	64.86%	75.14%	35.11%	36.65%	33.62%	22.67%	3.25%
Cyanobacteria/Chloroplast	13.19%	56.57%	77.22%	0.54%	50.39%	42.55%	29.66%	9.51%
Chloroflexi	18.70%	27.80%	74.43%	9.53%	51.08%	57.66%	31.06%	3.76%
Verrucomicrobia	19.81%	69.23%	71.92%	12.72%	7.52%	49.20%	30.26%	4.28%
Planctomycetes	17.82%	23.89%	75.58%	6.17%	53.70%	39.88%	33.25%	6.59%
Spirochaetes	8.50%	18.47%	57.74%	43.51%	42.69%	37.14%	31.16%	5.32%
Fusobacteria	6.29%	80.23%	80.80%	74.06%	64.01%	37.74%	17.57%	0.36%
Tenericutes	14.94%	51.38%	59.69%	49.00%	8.62%	49.78%	42.94%	0.50%
Nitrospira	14.16%	31.37%	52.51%	69.38%	59.93%	58.89%	23.53%	4.80%
TM7	9.92%	1.14%	74.52%	3.63%	3.00%	62.26%	19.33%	1.23%
Gemmatimonadetes	16.01%	77.80%	79.69%	70.39%	43.01%	41.07%	24.11%	5.18%
Deinococcus:Thermus	15.88%	77.29%	79.49%	0.58%	61.01%	61.59%	34.00%	0.98%
Chlorobi	24.25%	36.52%	67.90%	72.66%	56.74%	55.28%	34.55%	5.67%
Synergistetes	31.94%	64.23%	79.61%	0.84%	62.51%	60.64%	43.44%	24.42%
Armatimonadetes	17.70%	53.04%	71.47%	20.99%	48.69%	49.92%	22.45%	3.52%
Aquificae	10.85%	74.52%	79.38%	0.68%	26.61%	42.54%	29.15%	2.32%
Deferribacteres	10.24%	88.15%	88.15%	71.75%	38.67%	36.02%	25.12%	2.84%
Chlamydiae	0.82%	2.16%	1.34%	58.48%	0.82%	67.93%	42.75%	1.85%
Fibrobacteres	16.56%	51.32%	51.54%	48.14%	42.21%	32.13%	16.89%	1.32%
Thermotogae	16.49%	50.13%	85.45%	0.52%	63.38%	75.84%	40.78%	4.81%
SR1	12.52%	88.75%	88.75%	35.39%	0.73%	0.00%	0.18%	0.18%
OD1	19.70%	1.13%	4.69%	0.38%	0.94%	10.32%	0.75%	0.00%
Lentisphaerae	19.22%	70.96%	80.57%	64.01%	62.17%	49.49%	42.54%	4.29%
WS3	18.16%	40.13%	74.44%	16.59%	47.09%	28.92%	27.13%	3.59%
Elusimicrobia	13.41%	56.42%	82.40%	69.83%	76.26%	69.27%	31.01%	3.07%
OP11	20.88%	20.48%	20.48%	0.40%	34.94%	3.21%	22.49%	0.80%
BRC1	15.08%	57.54%	88.27%	5.59%	49.16%	48.04%	36.87%	3.91%
Thermodesulfobacteria	7.39%	80.11%	83.52%	0.57%	74.43%	73.86%	32.39%	0.00%
Caldiserica	18.18%	1.82%	3.03%	76.97%	59.39%	67.88%	26.67%	0.61%
Dictyoglomi	4.55%	2.27%	75.00%	0.00%	59.09%	63.64%	54.55%	20.45%
Chrysiogenetes	44.44%	100.00%	100.00%	100.00%	77.78%	77.78%	88.89%	55.56%
**Total**	11.58%	68.47%	76.01%	59.45%	56.53%	54.52%	22.08%	4.08%

Table S1 - *In silico* search for bacterial species coverage using RDP Database

Brocadiaceae	530f
Asahi BRW2	100.0%
Candidatus Brocadia	92.3%
Candidatus Jettenia	90.0%
Candidatus Kuenenia	100.0%
Candidatus Scalindua	95.7%
G1	100.0%
PB79	100.0%
W4(100%)	100.0%

Table S2 - *In silico* search for anammox bacteria coverage using SiLVA Database

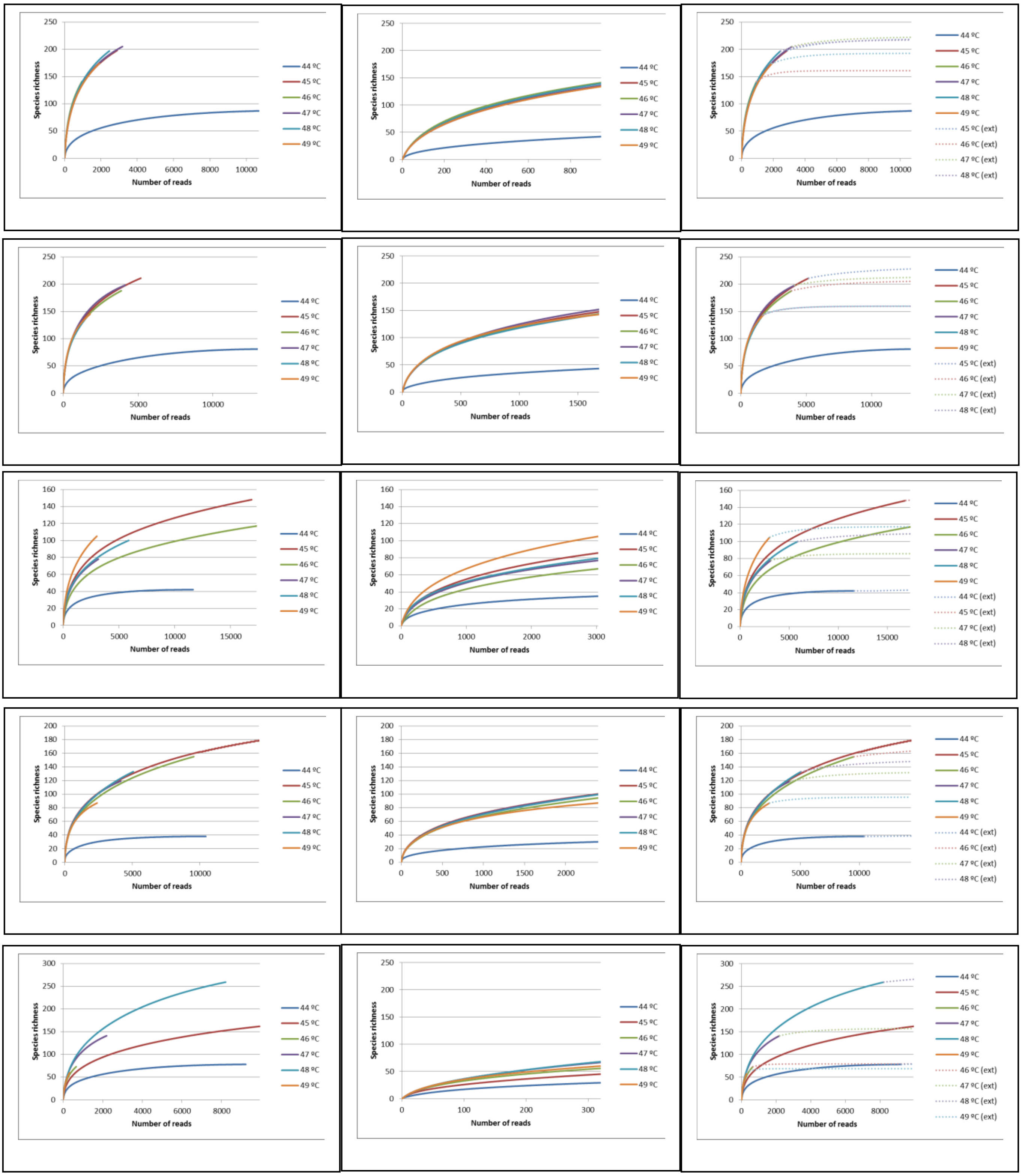

Figure S1 - Original (left column), interpolated to the lowest number of reads for each bioreactor (center column) and extrapolated to the highest number of reads for eachr bioreactor (right column) for the bioreactors analyzed in the study Lab MBR (first row), Low Temperature CANON (second row), A (third bioreactor), N (fifth bioreactor) and R (fifth bioreactor).

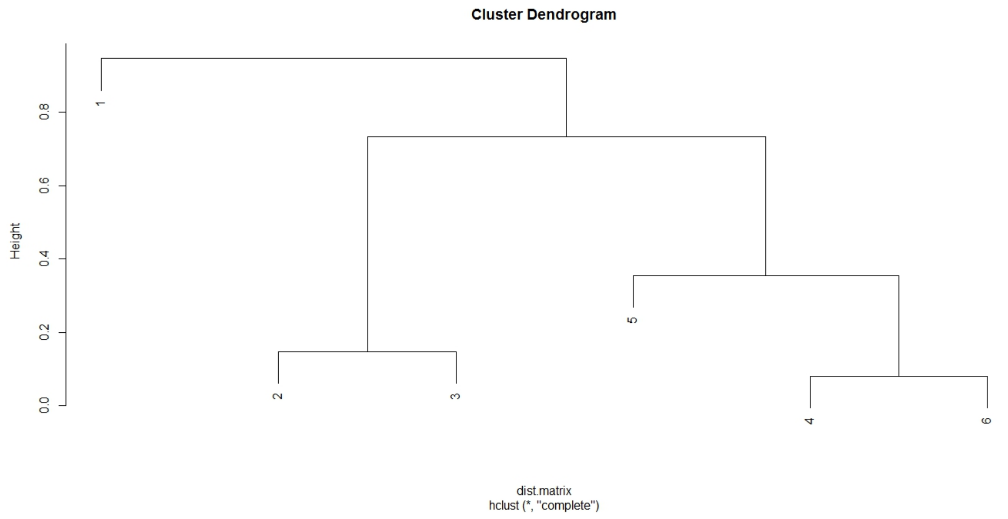

Figure S2 a) - Cluster analysis a) of bioreactor Lab MBR

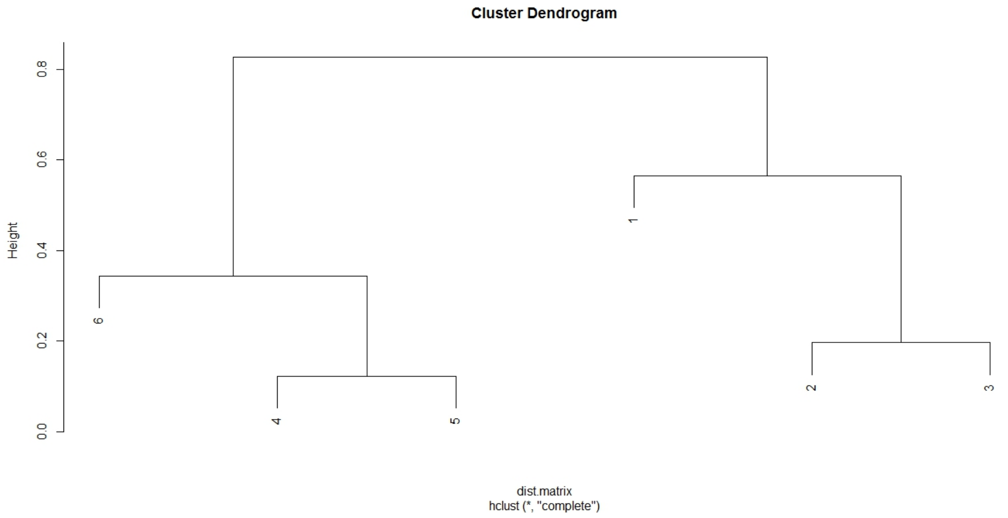

Figure S2 b) - Cluster analysis a) of bioreactor Low Temperature CANON

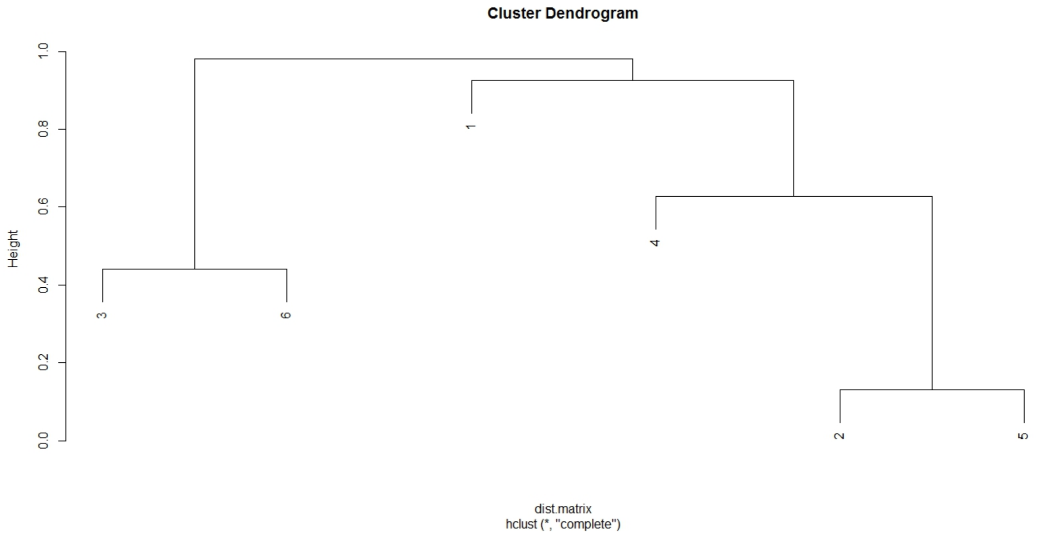

Figure S2 c) - Analysis cluster a) of bioreactor A

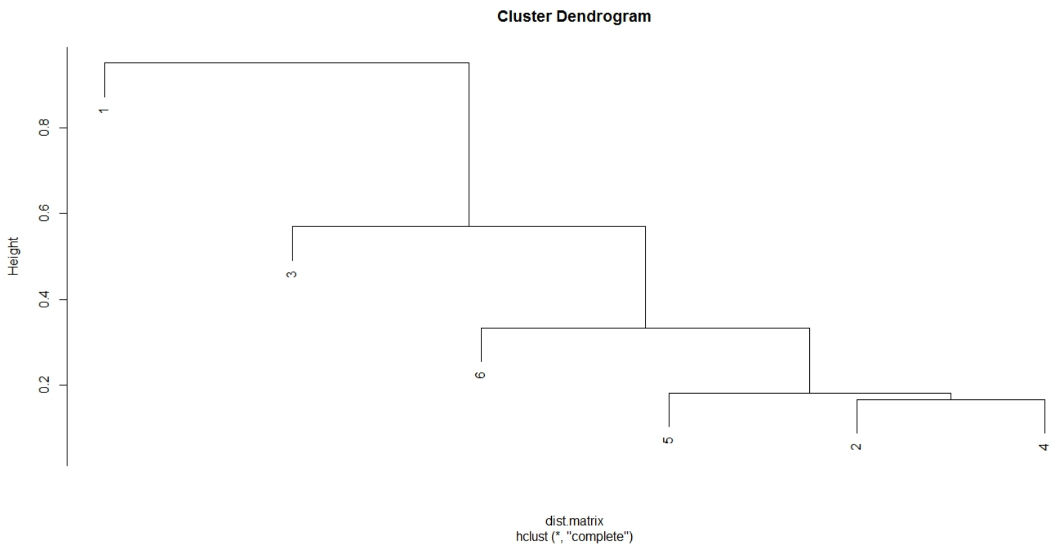

Figure S2 d) - Analysis cluster a) of bioreactor N

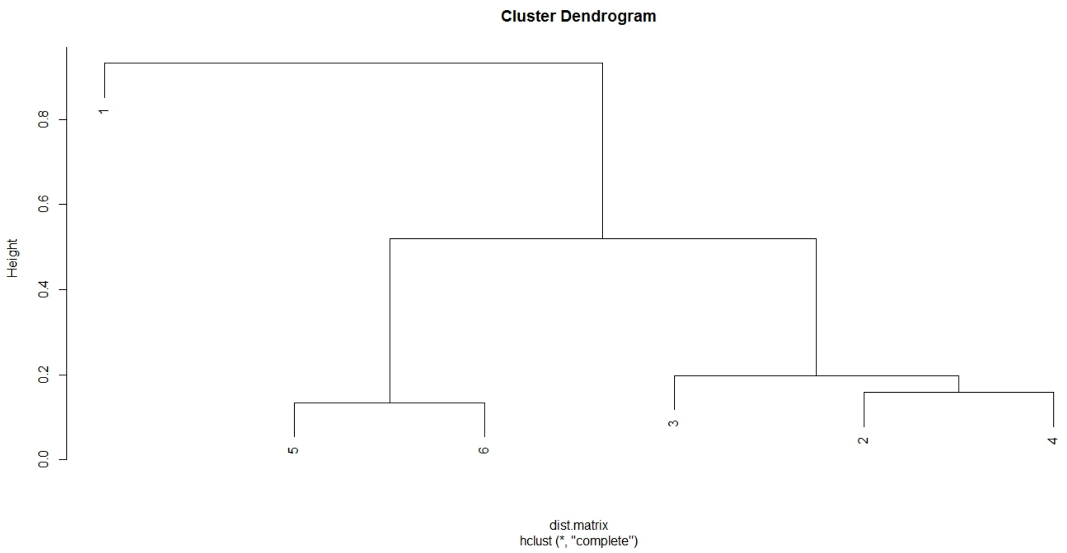

Figure S2 e) - Analysis cluster a) of bioreactor R

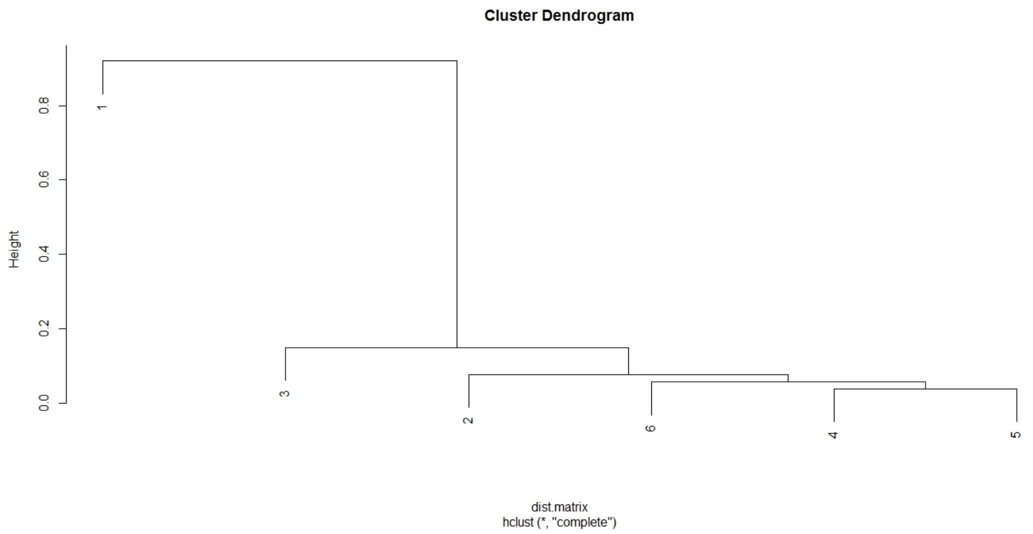

Figure S2 f) - Analysis cluster b) of bioreactor Lab MBR

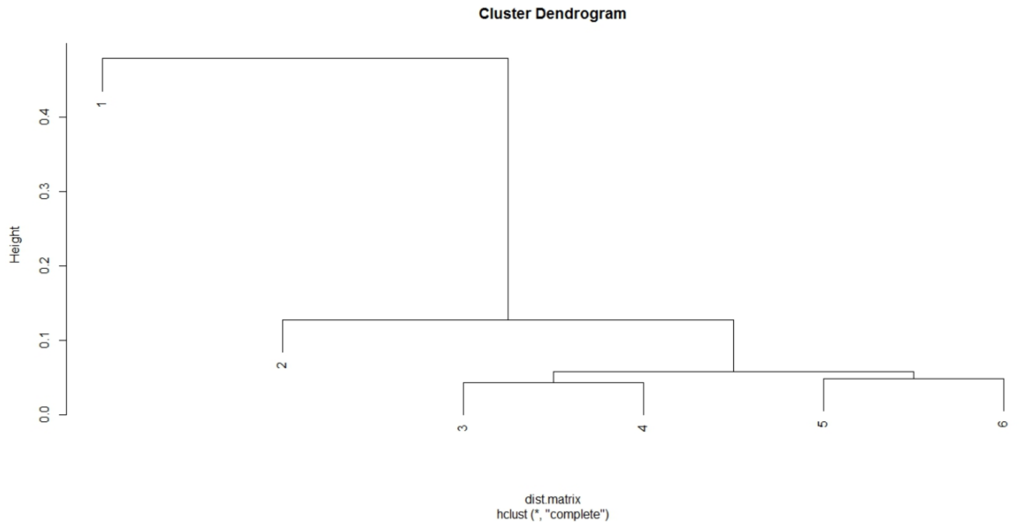

Figure S2 g) - Analysis cluster b) of bioreactor Low Temperature CANON

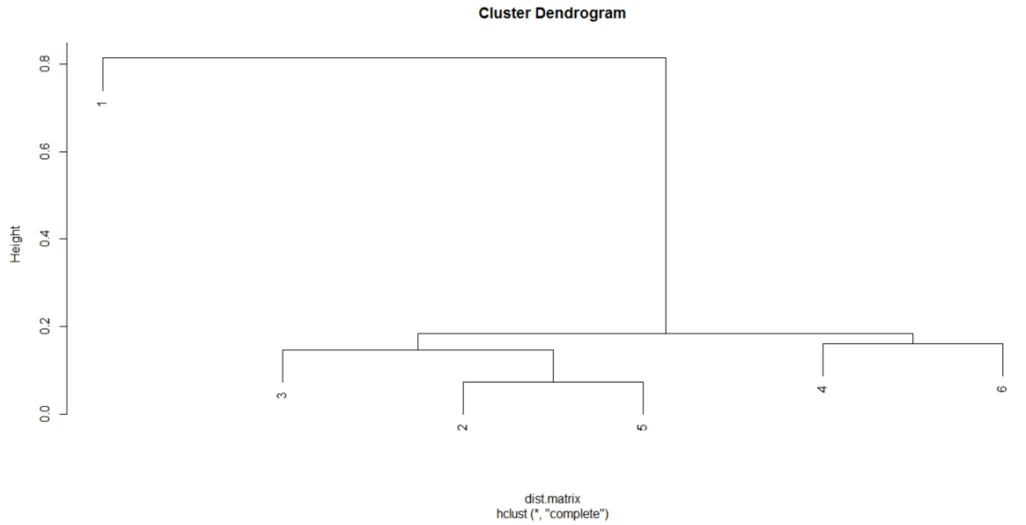

Figure S2 h) - Analysis cluster b) of bioreactor A

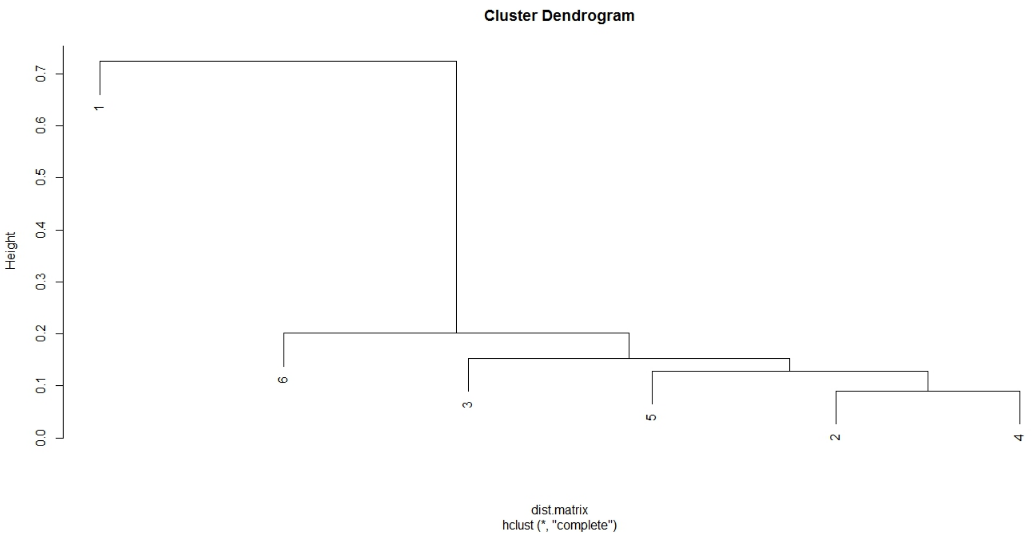

Figure S2 i) - Analysis cluster b) of bioreactor N

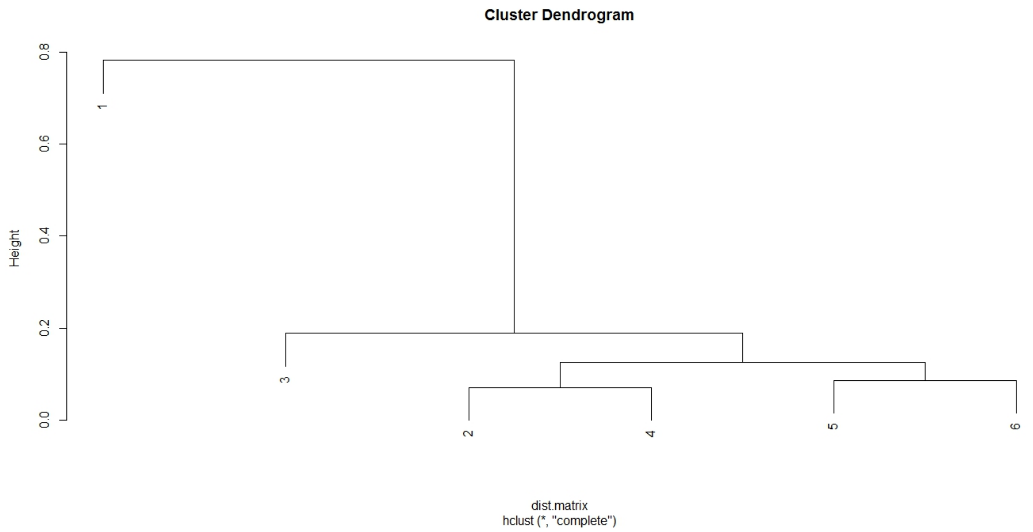

Figure S2 j) - Analysis cluster b) of bioreactor R

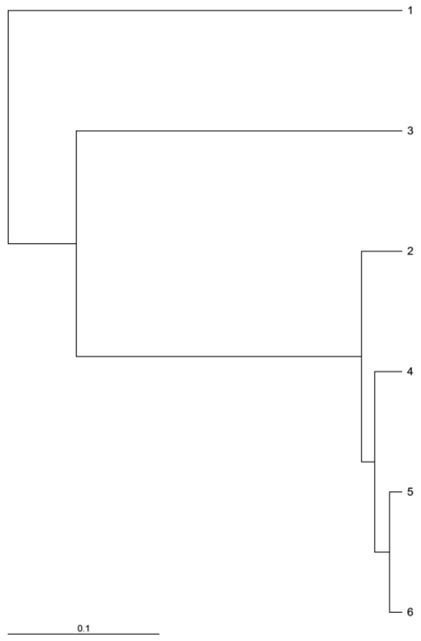

Figure S3 a) - Cluster analysis c) of bioreactor Lab MBR

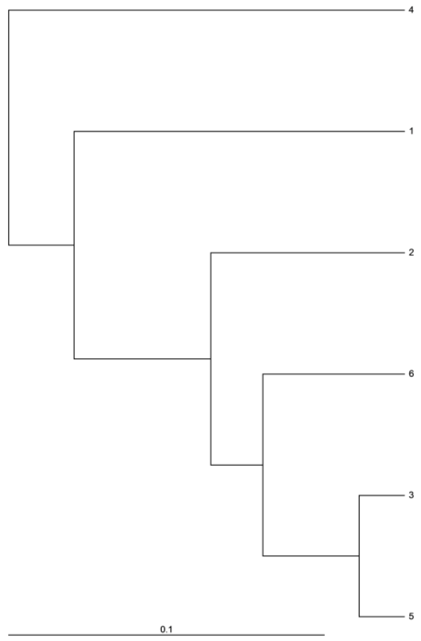

Figure S3 b) - Cluster analysis c) of bioreactor Low Temperature CANON

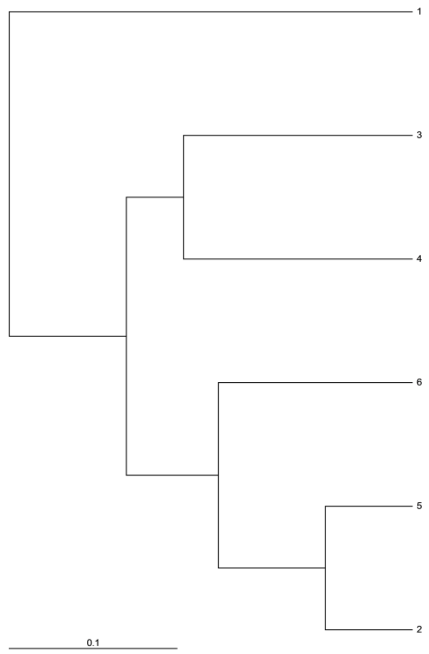

Figure S3 c) - Cluster analysis c) of bioreactor A

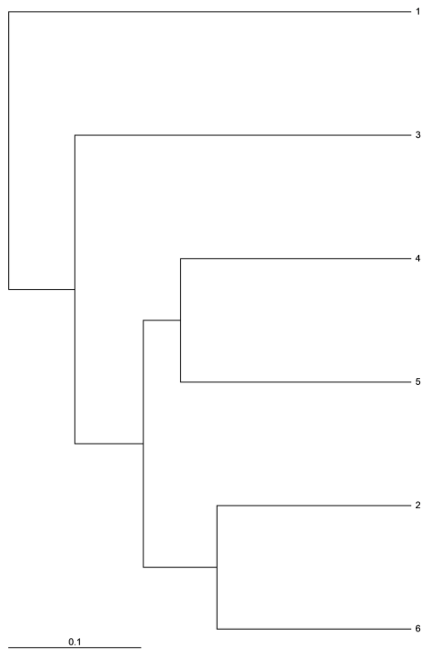

Figure S3 d) - Cluster analysis c) of bioreactor N

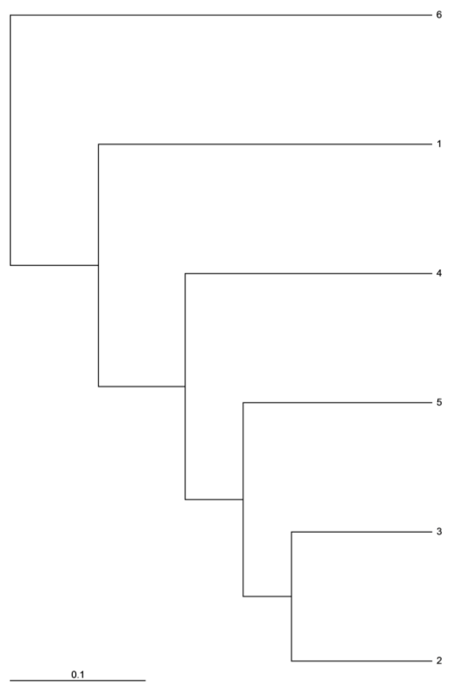

Figure S3 e) - Cluster analysis c) of bioreactor R

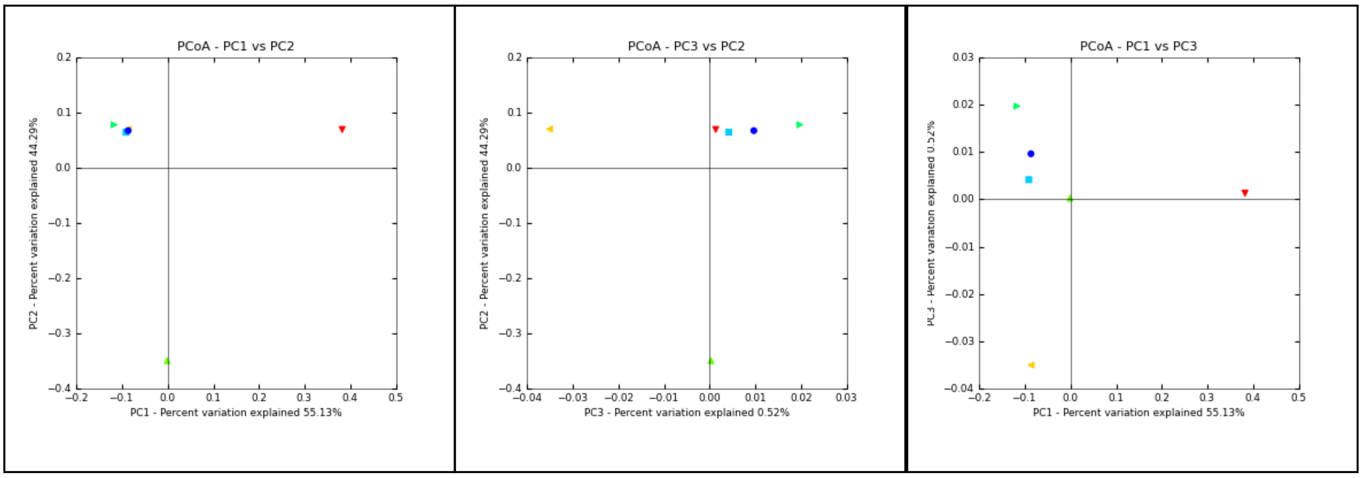

Figure S4 a) - Principal coordinates analysis of bioreactor Lab MBR

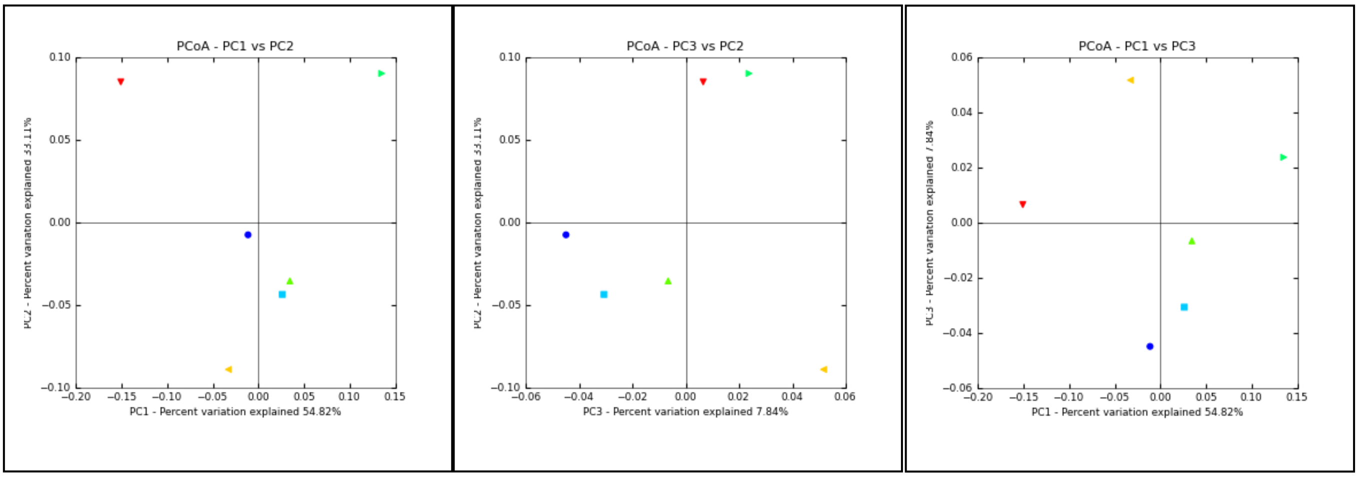

Figure S4 b) - Principal coordinates analysis of bioreactor Low Temperature CANON

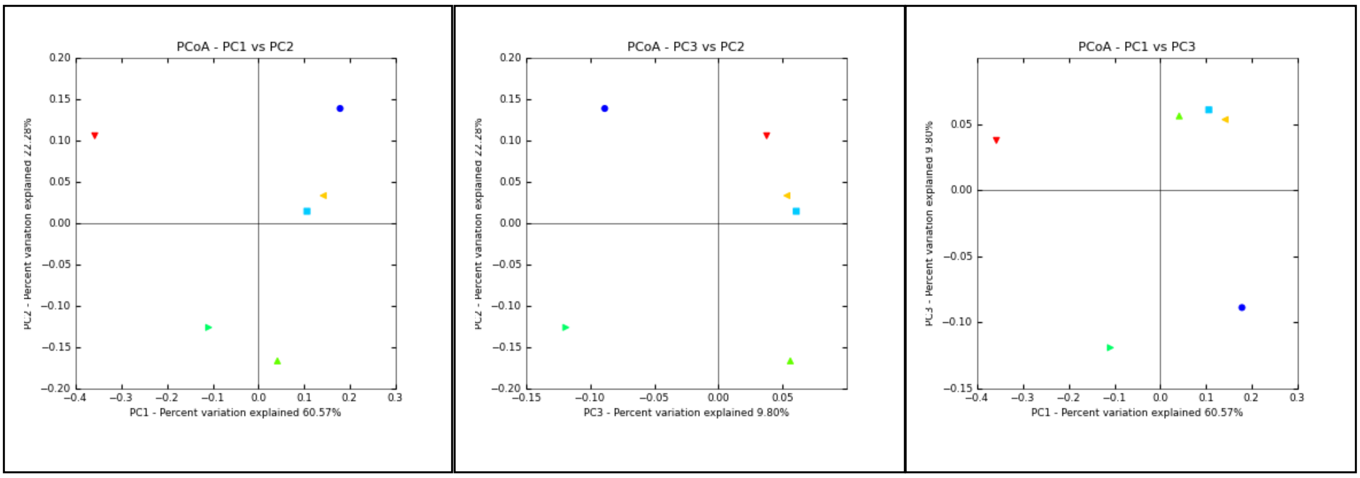

Figure S4 c) - Principal coordinates analysis of bioreactor A

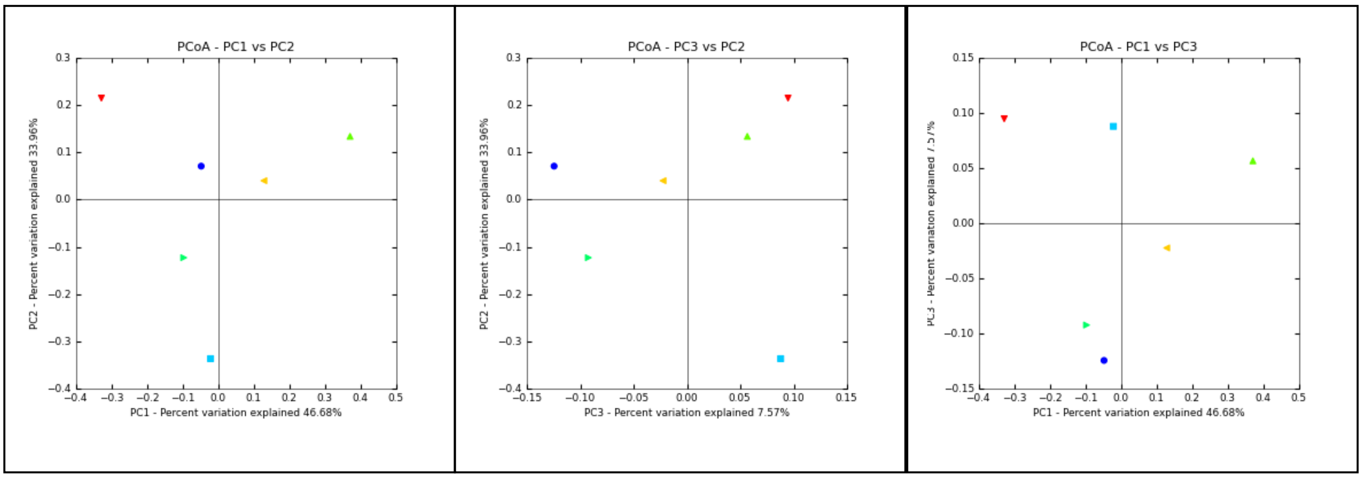

Figure S4 d) - Principal coordinates analysis of bioreactor R

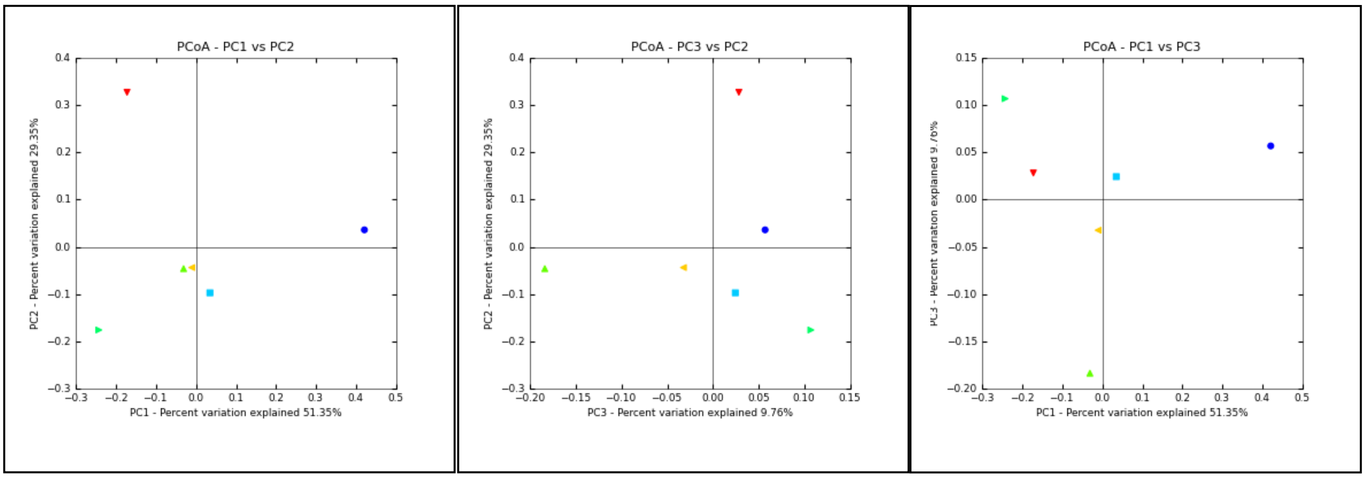

Figure S4 e) - Principal coordinates analysis of bioreactor N
